# Cerebral Embolic Protection in Transcatheter Aortic Valve Replacement

**DOI:** 10.1016/j.shj.2023.100169

**Published:** 2023-04-17

**Authors:** Mina Iskander, Yasser Jamil, John K. Forrest, Mahesh V. Madhavan, Raj Makkar, Martin B. Leon, Alexandra Lansky, Yousif Ahmad

**Affiliations:** a Yale School of Medicine, Yale University, New Haven, Connecticut, USA; b Columbia University Irving Medical Center/New York-Presbyterian Hospital, New York, New York, USA; c Cardiovascular Research Foundation, New York, New York, USA; d Smidt Heart Institute, Cedars-Sinai Medical Center, Los Angeles, California, USA; e Barts Heart Centre, London and Queen Mary University of London, London, UK; f Department of Cardiovascular Medicine, Medical College of Wisconsin, Milwaukee, Wisconsin

**Keywords:** Aortic stenosis, Cerebral protection, Stroke, TAVR

## Abstract

Transcatheter aortic valve replacement (TAVR) is a treatment option for patients with symptomatic severe aortic stenosis across the entire spectrum of surgical risk. Recent trial data have led to the expansion of TAVR into lower-risk patients. With iterative technological advances and successive increases in procedural experience, the occurrence of complications following TAVR has declined. One of the most feared complications remains stroke, and patients consider stroke a worse outcome than death. There has therefore been great interest in strategies to mitigate the risk of stroke in patients undergoing TAVR. In this paper, we will discuss mechanisms and predictors of stroke after TAVR and describe the currently available cerebral embolic protection devices, including their design and relevant clinical studies pertaining to their use. We will also review the current overall evidence base for cerebral embolic protection during TAVR and ongoing randomized controlled trials. Finally, we will discuss our pragmatic recommendations for the use of cerebral embolic protection devices in patients undergoing TAVR.

## Graphical abstract

**Figure:**
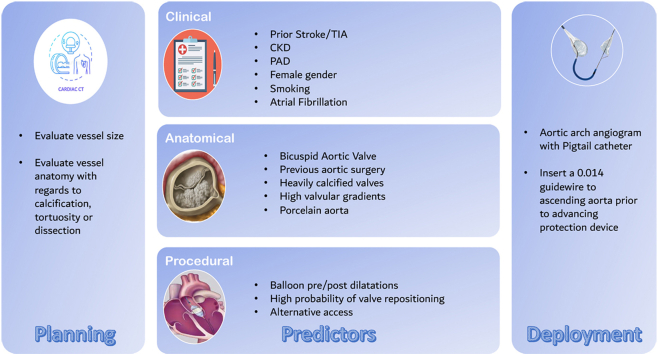

Graphical Abstract. Summary of predictors of cerebrovascular events for patients undergoing transcatheter aortic valve replacement and considerations for planning and deploying cerebral embolic protection devices during transcatheter aortic valve replacement.

## Introduction

Transcatheter aortic valve replacement (TAVR) has become a treatment of choice for symptomatic severe aortic stenosis (AS) across the spectrum of surgical risk,^1^, ^2^, ^3^, ^4^, ^5^ with recent expansion to younger and lower-risk patients.^6^^,^^7^ Technological and experiential advancements have addressed potential complications of TAVR such as paravalvular regurgitation and new pacemaker implantation and seen the occurrence of these steadily decline.

A feared complication of TAVR, or indeed any cardiac intervention or surgery, is stroke. Patients consider avoidance of stroke as a more important outcome than survival,^8^^,^^9^ and patients undergoing TAVR specifically value maintaining independence above simply staying alive.^10^ Strategies to reduce the occurrence of stroke are therefore of great interest. The incidence of post-TAVR stroke ranged from 5% to 6% in the initial pivotal trials, with declining rates to ∼1% to 2% seen in the most recent clinical trials (see Table 1). These declining rates likely reflect a combination of improvements and advancements in TAVR technology, increasing operator experience, and sequentially lower-risk patients being treated. In real-world registries, stroke rates have remained constant at 2% to 2.5%,^11^^,^^12^ but the occurrence of stroke after TAVR is associated with a significant impact on long-term morbidity and mortality.^11^ In a recent analysis of the Society of Thoracic Surgeons/American College of Cardiology Transcatheter Valve Therapies (TVT) Registry, the occurrence of stroke within 30 days after TAVR was associated with increased risk-adjusted 30-day mortality (hazard ratio, 6.1; 95% CI, 5.4-6.8; *p* < 0.001).^11^ Strategies to prevent stroke after TAVR are therefore desired and have led to the development of several devices designed to protect against stroke. In this review, we will discuss mechanisms and predictors of stroke after TAVR and the currently available cerebral embolic protection devices (CEPDs), including their design and relevant clinical studies pertaining to their use. We will also review the current overall evidence base for cerebral embolic protection during TAVR and ongoing randomized controlled trials (RCTs). Finally, we will discuss our pragmatic recommendations for the use of CEPDs in patients undergoing TAVR.

**Table 1:** Incidence of stroke in pivotal trials of transcatheter aortic valve replacement

Study	Treatment groups	Study population	30-d stroke rate (%)	1-y stroke rate (%)
PARTNER 1, Cohort B 2010	TAVR vs. OMT	Inoperable	5.0 vs. 1.1 (*p* = 0.06)	7.8 vs. 3.9 (*p* = 0.18)
PARTNER 1, Cohort A 2011	TAVR vs. SAVR	High risk	3.8 vs. 2.1 (*p* = 0.20)	5.1 vs. 2.4 (*p* = 0.07)
CoreValve Extreme Risk 2014	TAVR (single arm)	Inoperable	2.3	4.3
CoreValve High Risk 2014	TAVR vs. SAVR	High risk	3.9 vs. 3.1 (*p* = 0.55)	5.8 vs. 7.0 (*p* = 0.59)
PARTNER 2 A 2016	TAVR vs. SAVR	Intermediate risk	3.2 vs. 4.3 (*p* = 0.20)	5.0 vs. 5.8 (*p* = 0.46)
SURTAVI∗ 2017	TAVR vs. SAVR	Intermediate risk	1.2 vs. 2.5 (−2.6, 0.1)	2.2 vs. 3.6 (−3.1. 0.4)
PARTNER 3 2019	TAVR vs. SAVR	Low risk	0.6 vs. 2.4 (*p* = 0.02)	1.2 vs. 3.3 (*p* = 0.03)
EVOLUT Low Risk Trial∗ 2019	TAVR vs. SAVR	Low risk	3.4 vs. 3.4 (−1.9, 1.9)	4.1 vs. 4.3 (−2.4, 1.9)

EVOLUT, transcatheter aortic valve replacement with a self-expanding valve; OMT, optimal medical therapy; PARTNER, the placement of aortic transcatheter valves; SAVR, surgical valve replacement; SURTAVI, surgical replacement and transcatheter aortic valve implantation; TAVR, transcatheter aortic valve replacement.

∗ Values are posterior median rates and 95% credible interval for the difference between groups, all results reported as modified intention-to-treat analysis unless otherwise indicated.

## Silent Cerebrovascular Events After TAVR

Several studies have shown that patients undergoing TAVR have new silent cerebral ischemic embolic lesions, affecting both cerebral hemispheres in most patients.^13^^,^^14^ These are detected by diffusion-weighted magnetic resonance imaging (DW-MRI) as hyperintense signals. Studies have reported that brain lesions were found in 94% of patients who underwent DW-MRI, and these lesions appear to have clinical significance in terms of functional neurological status with 41% of patients demonstrating cognitive decrements at 30 days.^15^ In the Rotterdam Scan Study, silent cerebral infarction was associated with a threefold increased risk of stroke, greater decline in cognitive function, and a 2-fold increased risk of dementia after a 4-year follow-up.^16^

## Mechanism of Cerebrovascular Events

Strokes can potentially be underdiagnosed after TAVR due to the absence of a standardized definition and classification. In view of this, the Valve Academic Research Consortium has introduced consensus documents outlining uniform endpoint definitions.^17^^,^^18^ Stroke is defined as a new neurological deficit with a duration of ≥24 ​hours, or <24 ​hours associated with cerebral injury on a neuroimaging study. A transient ischemic attack (TIA) is defined as a duration of a focal or global neurological deficit <24 ​hours, with neuroimaging not demonstrating a new hemorrhage or infarct. Strokes may be classified as ischemic or hemorrhagic.^18^ The severity of stroke is usually categorized according to the modified Rankin Scale (mRS), classifying it into disabling (major stroke mRS ≥2) and non-disabling (minor stroke mRS <2).

Cerebrovascular events have been also classified according to their temporal pattern as acute (≤24 ​hours), sub-acute (1-30 days), and late (>1 month) events. Most strokes usually occur early after the TAVR procedure.^19^, ^20^, ^21^ In their analysis of 2621 patients in the Placement of Aortic Transcatheter Valves (PARTNER) 1 trial, Kapadia and colleagues^22^ noted that 85% of strokes occurred within 1 week after TAVR with a peak instantaneous risk on day 2.

The majority of post-TAVR strokes are ischemic.^12^ Most cerebrovascular events are thought to be caused by emboli released from the manipulation of atherosclerotic debris by catheters, guidewires, balloons, or during the implantation of the valve within severely calcified native aortic leaflets. This mechanism is supported by transcranial doppler studies, which use high-intensity signals as a surrogate for microembolization and have demonstrated these in the middle cerebral arteries during phases of the procedure, especially during valve positioning and implantation.^13^^,^^23^ This was also confirmed by histopathological studies, where debris consisting of arterial wall tissue, calcifications, native valve tissue, and foreign material was found in filters in up to 99% of examined patients.^24^^,^^25^ These findings underpin the rationale for using CEPDs for the prevention of stroke during TAVR.

The etiology of late cerebrovascular events, occurring more than 48 ​hours after TAVR, is still not well established. The primary cause is thought to be thromboembolic related to thrombus formation on the TAVR prosthesis,^26^^,^^27^ new-onset atrial fibrillation,^28^ or dislodged particles that later embolize with increased flow.

## Predictors of Cerebrovascular Events After TAVR

Several studies have reported on predictors of developing cerebrovascular events after TAVR.^19^^,^^20^^,^^22^^,^^29^, ^30^, ^31^ These factors can be broadly divided into early (acute and subacute) and late events.

Predictors of early stroke can include both patient and procedural factors. Procedural risk factors for early stroke included more post-dilatations and possibly more pacing runs, as well as higher pre-TAVR peak transaortic gradient (possibly reflecting more severe stenosis with more calcium, or requiring more instrumentation to cross and complete the procedure).^22^ These data align with the findings reported by Nombela-Franco et al.,^20^ where in a cohort of 1061 patients undergoing TAVR, patients with a higher degree of valve calcification more frequently underwent balloon post-dilation which was itself found to be a strong predictor for early cerebrovascular events (odds ratio [OR], 2.46; 95% CI,1.07-5.67). In a large meta-analysis including >72,000 patients, female sex, chronic kidney disease, the performance of TAVR during the first half of centers’ experience, and new-onset atrial fibrillation were associated with an increased risk of early cerebrovascular events.^32^ However, valve type (balloon-expandable vs. self-expandable) and approach (transfemoral vs. non-transfemoral) did not predict cerebrovascular events. In a recent analysis of the TVT registry conducted by Thourani et al including 97,600 patients, alternative access for TAVR (i.e., utilization of access other than transfemoral and transaortic) had the greatest OR for in-hospital stroke.^31^ Transaortic access was also an independent predictor; although some other studies have shown comparable rates of stroke after transaortic and transfemoral TAVR.^13^^,^^19^^,^^33^ Other predictors of in-hospital stroke post-TAVR include prior stroke, prior TIA, preprocedural shock, use of inotropes or mechanical assist devices, smoking, porcelain aorta, peripheral arterial disease, and prior aortic valve and non-aortic valvular procedures.^31^ Data regarding stroke in valve-in-valve (ViV) TAVR are scarce. However, in a recent review comparing the risk for stroke and mortality rates in ViV TAVR procedures with native TAVR approach, a quantitative analysis showed no significant differences in 30-day stroke rates when comparing ViV TAVR to either native TAVR or surgery.^34^

Predictors of late stroke are mainly related to a patient’s baseline atherosclerotic risk and frailty. In the PARTNER trial, investigators noted that dementia and a smaller prosthetic valve size (23 vs. 26 mm) were predictors for late stroke in the transfemoral cohort; and race (non-White), lower left ventricular ejection fraction, and atrial fibrillation, for the transapical approach.^22^ In the CoreValve trials, small body surface area, severe aortic calcification, and falls within the past 6 ​months were significant predictors for late stroke.^29^

## Cerebral Embolic Protection Devices

Due to the risk of stroke following TAVR, there has been interest in strategies to reduce this procedural hazard. CEPDs are devices deployed during the TAVR procedure to protect against embolism to the brain and consequent stroke and can be broadly classified into filters or deflectors (see Table 2). Filters achieve cerebral protection by capturing and extracting emboli from the circulation while deflectors alternate the route of the emboli away from the cerebral circulation to the systemic circulation. The actual efficacy of the device depends on the capacity to protect the 3 main branches of the aortic arch, filter size, and the ability to deploy without disrupting aortic arch plaque (which can itself lead to a risk of atheroembolism and stroke). Currently, there are 4 devices available with clinical studies evaluating their safety and efficacy (see Table 3).

**Table 2:** Characteristics of current cerebral embolic protection devices

Device	Manufacturer	Design	Access	Delivery system	Neurological coverage
SENTINEL™	Boston Scientific, Marlborough, Massachusetts, United States	Filter	Radial artery	6 Fr	Partial
TriGuard	Keystone Heart Ltd, Caesarea, IL, USA	Deflector	Femoral artery	8-9 Fr	Complete
Embrella	Edwards Lifesciences, Irvine, California, United States	Deflector	Radial artery	6 Fr	Partial
Embol-X	Edwards Lifesciences, Irvine, California, United States	Filter	Direct Aortic	14 Fr	Complete

Images reproduced with permission from Vlastra W, Vendrik J, Koch KT, Baan J, Piek JJ, Delewi R. Cerebral protection devices during transcatheter aortic valve implantation. Trends Cardiovasc Med. 2018 Aug; 28(6):412-418.

Fr, French.

**Table 3:** Current clinical trial evidence for cerebral embolic protection devices

Device	Studies/authors	Study design	Number of participants	Neuroimaging results	Clinical outcome results	Complications
SENTINEL™	MISTRAL-C—Van Mieghem et al., 2016^35^	RCT	Device: 32 Control: 33	Median total new lesion volume 5 d post TAVR: 95 mm3 vs. 197 mm3 (*p* = 0.17)	Stroke disabling 30 d (%): 0 vs. 7	Major vascular complication (%): 0 vs. 19
	CLEAN-TAVI—Haussig et al., 2016^36^	RCT	Device: 50 Control: 50	Median total new lesion volume 2 d post TAVR: 242 mm3 vs. 527 mm3 (*p* = 0.001)	Stroke non-disabling 7 d (%): 10 vs. 10	Major vascular complication (%): 10 vs. 12
	SENTINEL—Kapadia et al., 2017^25^	RCT	Device: 121 Control: 119 Safety: 123	Median total new lesion volume 2-7 d post TAVR: 102.83 mm3 vs. 177.98 mm3 (*p* = 0.33)	MACCE 30 d (%): 7.3 vs. 9.9 (*p* = 0.4); Stroke 30 d (%): 5.6 vs. 9.1 (*p* = 0.25)	Major vascular complication (%): 5.9 vs. 8.6 (*p* = 0.53)
	PROTECTED TAVR—Kapadia et al., 2022^37^	RCT	Device: 1501 Control: 1499	Using the Neurologic Academic Research Consortium (NeuroARC) to classify the strokes into type 1.a to 1.d and type 2.b. Stroke size was not reported.	Stroke (%): 2.3 vs. 2.9 (*p* = 0.30), disabling stroke: 0.5 vs. 1.3%; ​(*p* = 0.02), nondisabling stroke: 1.7 vs. 1.5; ​(*p* = 0.67), mortality (0.5 vs. 0.3)	Major or minor vascular complication (%), bleeding: 0.1 vs. 0
TriGuard	DEFLECT III—Lansky et al., 2015^38^	RCT	Device: 46 Control: 39	Freedom from ischemic brain lesions 30 d post TAVR: 26.9 vs. 11.5	MACCE composite in hospital (%): 21.7 vs. 30.8 (*p* = 0.34); Stroke composite in hospital (%): 2.2 vs. 5.1 (*p* = 0.46)	Major vascular complication (%): 15.2 vs. 15.4 (*p* = 0.85)
	REFLECT I—Lansky et al., 2020^39^	RCT	Device: 141 Control: 63 Roll-in: 54	Median total new lesion volume 2-5 d post TAVR: 229 mm3 vs. 235 mm3 (*p* = 0.885)	MACCE 30 d (%): 21.8 vs. 8.5 RR 2.57 (1.05-6.32; Stroke 30 d (%): 10.7 vs. 6.8 RR 1.58 (0.54-4.59)	Major vascular complication (%): 12.3 vs. 1.7 (*p* = 0.025)
	REFLECT II—Nazif et al., 2021^40^	RCT	Device: 162 Control: 121	Median total new lesion volume 2-5 d post TAVR: 215.4 mm3 vs. 188.1 mm3 (*p* = 0.40)	MACCE 30 d (%): 15.9 vs. 7 (*p* = 0.115); Stroke 30 d (%): 6.4 vs. 5.3 (*p* = 1.0)	Major vascular complication (%): 7.0 vs. 0 (*p* = 0.04)
Embrella	PROTAVI-C—Rodes-Cabau et al., 2014^33^	Observational	Device: 41 Control: 11	Median total new lesion volume 7 d post TAVR: 305 mm3 vs. 180 mm3 (*p* = 0.9)	Stroke 30 d (%): 4.9 vs. 0 (*p* = 1.0)	Major vascular complication (%): 12.2 vs. 9.1 (*p* = 1.0)
Embol-X	Embol-X—Wendtl et al., 2015^41^	RCT	Device: 14 Control: 16	Median total new lesion volume 7 d post TAVR: 88 ± 60 vs. 168 ± 217 (*p* = 0.27)	Stroke 30 d (%): 0 vs. 0	NA

CLEAN-TAVI, the Claret embolic protection and TAVI trial; MACCE, major adverse cardiovascular and cerebrovascular event; MISTRAL-C, MRI Investigation in TAVI with Claret trial; PROTAVI-C, the prospective randomized outcome study in patients undergoing TAVI to examine cerebral ischemia and bleeding complications trial; PROTECTED TAVR, stroke protection with Sentinel during transcatheter aortic valve replacement; RCT, randomized controlled trial; RR, relative risk; TAVR, transcatheter aortic valve replacement.

### SENTINEL

**Figure 1:**
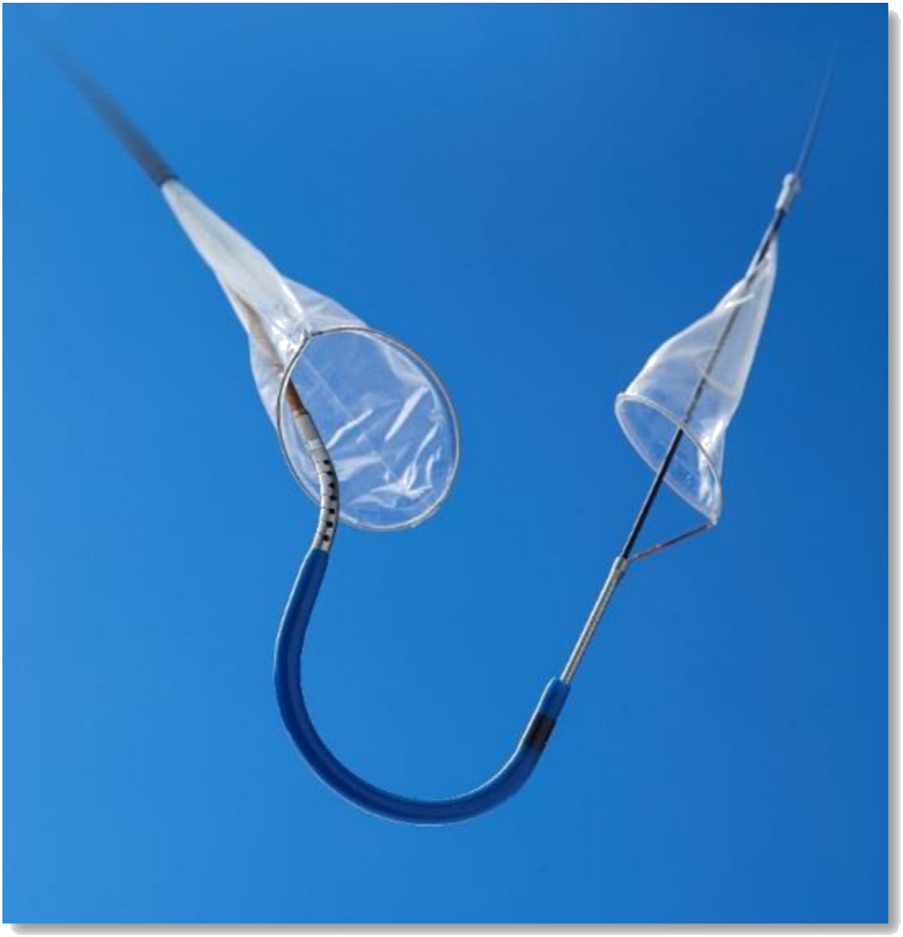
**Sentinel device with 2 independent polyurethane filters**.

The Sentinel cerebral protection system (Boston Scientific, Marlborough, Massachusetts) is the most widely-studied and utilized system, and received approval by the United States Food and Drug Administration in 2017 and the Conformité Européene mark in 2013. The device consists of 2 interconnected filters within a 6 French (Fr) delivery system (see Figure 1). The proximal filter measures 9 to 15 mm and is delivered in the brachiocephalic trunk while the distal filter measures 6.5 to 10 mm and is delivered in the left common carotid artery. The left vertebral artery, which usually originates from the left subclavian artery, remains unprotected. The device is placed percutaneously via the right radial/brachial artery over a 0.014-inch coronary guidewire. A review of an available TAVR computed tomography scan prior to the procedure is advisable to evaluate if the anatomy of the aortic arch and its branches is suitable and to rule out excessive tortuosity and calcification. The Sentinel device has to date been studied in 4 RCTs, MRI Investigation in TAVI with Claret (MISTRAL-C) trial, The Claret Embolic Protection and TAVI (CLEAN-TAVI) trial, SENTINEL trial, and the Stroke Protection with Sentinel During Transcatheter Aortic Valve Replacement (PROTECTED-TAVR) trial.^25^^,^^35^^,^^36^

The MISTRAL-C trial^35^ was the first multicenter double-blind RCT that evaluated the efficacy and safety of the Sentinel device. A total of 65 patients were randomized 1:1 to transfemoral TAVR with or without the Sentinel device. The goal was to determine whether cerebral protection of the SENTINEL device would reduce the incidence of new ischemic brain lesions as assessed by DW-MRI and prevent neurocognitive decline, as assessed with the Montreal Cognitive Assessment and the Mini-Mental State Examination. Patients undergoing transfemoral TAVR were randomized 1:1 to cerebral protection with Sentinel or control (no Sentinel). The magnetic resonance imaging (MRI) scan and neurocognitive evaluations were performed 1 ​day before TAVR and planned for 5 to 7 days after TAVR. Patients randomized to receive the Sentinel device had numerically fewer new lesions and a smaller total lesion volume (95 mm^3^ [Interquartile range, 10-257] vs. 197 mm^3^ [95-525]) but neither reduction was statistically significant. It should be noted, however, that only 57% of the randomized patients underwent follow-up DW-MRI. Neurocognitive deterioration was present in 4% of patients with Sentinel device vs. 27% of patients without (*p* = 0.017). The filters captured debris in all patients with Sentinel device. The main limitation of this study is the small sample size and this was compounded by the high MRI dropout rate further reducing power. There were 2 disabling strokes in the entire study cohort, both occurring in the control arm and with both patients dying within 30 days. This trial was not powered for clinical endpoints, however.

The CLEAN TAVI trial^36^ was a single-center, blinded RCT that included 100 patients undergoing transfemoral TAVR who were randomized 1:1 to Sentinel or control. The primary endpoint was the number of new brain lesions within protected territories detected by DW-MRI 2 ​days after TAVR. The number of new lesions were significantly reduced in the device group when compared to the control group (4 vs. 10, *p* < 0.001), as was the median total new lesion volume (242 mm^3^ vs. 527 mm^3^, *p* = 0.001). 5 patients in each group suffered strokes, all non-disabling; once again this trial was underpowered for clinical events.

The SENTINEL trial^25^ was a multicenter RCT evaluating the safety and efficacy of cerebral protection during TAVR, and again assessing the Sentinel device. The study included 363 patients from 19 centers, randomized 1:1:1 into a safety arm (device use only, without imaging analysis), and 2 imaging cohorts, in which patients randomly underwent TAVR with or without the Sentinel device. The primary safety endpoint was major adverse cardiovascular and cerebrovascular events (MACCEs) at 30 days and the primary efficacy endpoint was new lesion volume detected by DW-MRI at 2 to 7 days. There was no statistically significant difference in the occurrence of MACCE in the device and control arms (7.3% vs. 9.9%, *p* = 0.4) or of strokes at 30 days (5.6% in the device arm vs. 9.1% in the control arm, *p* = 0.25). There was also no difference in new lesion volume on DW-MRI (102.83 mm^3^ in the device arm vs. 177.98 mm^3^ in the control arm, *p* = 0.33). Neurocognitive function was similar in both groups, but there was a correlation between lesion volume and neurocognitive decline (*p* = 0.0022). The device was associated with a favorable safety profile and debris was found within filters in 99% of patients.

The PROTECTED-TAVR trial is the largest randomized TAVR trial to date, evaluating the Sentinel CEPD during TAVR, with 3000 randomized patients from 51 centers, with 1:1 randomization between Sentinel CEPD and control (TAVR without CEPD). The primary endpoint was clinical stroke (classified according to the Neurologic Academic Research Consortium) that occurred within 72 ​hours of the TAVR procedure or before hospital discharge. The stroke was diagnosed by a neurology professional and supported by neuroimaging if stroke was suspected. CEPD was successfully placed in 94.4% of patients in whom it was attempted.^37^

There was no significant difference in the rate of the primary endpoint between the 2 groups: 2.3% in the CEPD group and 2.9% in the control group (difference, −0.6%; 95% CI, −1.7 to 0.5; *p* = 0.30). There was a significant reduction in the rate of disabling stroke with CEPD compared to control: 0.5% vs. 1.3%; difference, −0.8%; 95% CI, −1.5 to −0.1. The Sentinel CEPD device was shown to be safe, with a vascular access-site complication rate of 0.1% and no differences in the rates of acute kidney injury between the 2 groups.

### TriGUARD

**Figure 2:**
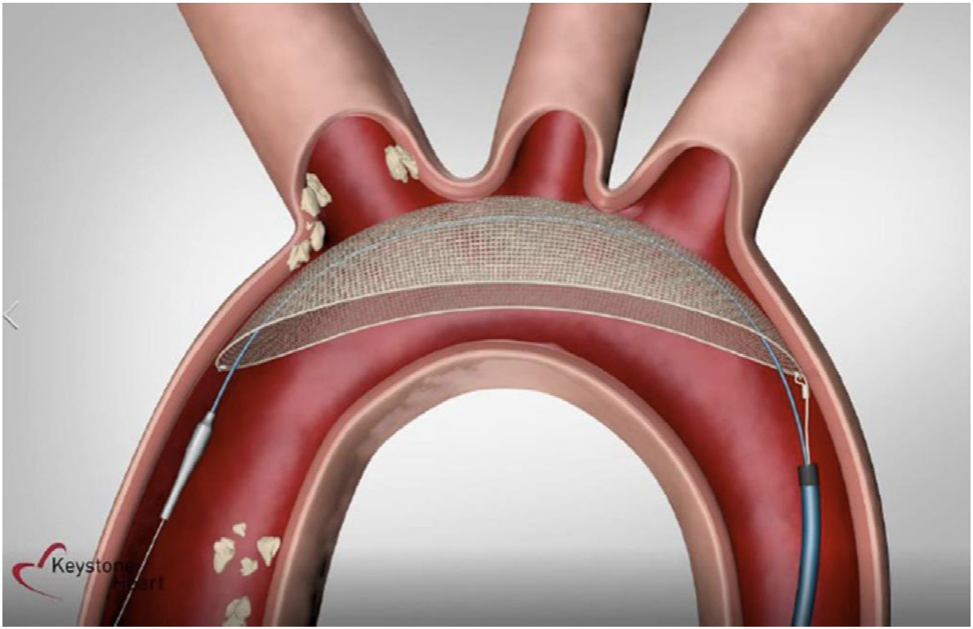
**TriGUARD 3 device positioned across aortic arch providing complete brain coverage**.

The TriGUARD HDH Embolic device (TG) (Keystone Heart Ltd, Caesarea, Illinois) is the second most studied device, and functions as a deflector device that offers complete brain protection by covering all 3 cerebral vessels in the aortic arch (see Figure 2). The system consists of a single-use filter made of a fine nickel-titanium alloy mesh, delivered through a contralateral 9 Fr femoral arterial sheath; this sheath can accommodate the pigtail catheter used during the TAVR procedure and therefore does not require additional vascular access. The TG device is positioned under fluoroscopic guidance to cover the ostia of 3 main aortic branches and anchored in place by a stabilizer positioned in the proximal innominate artery. TG has been studied in 3 RCTs: DEFLECT III, REFLECT I, and REFLECT II.^38^, ^39^, ^40^

The DEFLECT III trial^38^ was the first multi-center RCT to evaluate the efficacy and safety of the TG. The study included 85 patients at 13 centers; patients were randomized 1:1 to either cerebral protection with TG or control (no cerebral protection). In the intention-to-treat analysis, the device group was associated with a greater freedom from ischemic brain lesions at 30 days than the control group (26.9% vs. 11.5%). MACCE at 30 days occurred in 21.7% of patients in the device group, compared to 30.8% in the control group but this difference was not statistically significant (*p* = 0.34). There was also no significant difference in stroke at 30 days between the 2 groups (2.2% vs. 5.1%, *p* = 0.46). There were suggestions of improvement in neurocognitive function in the device group, although the differences were not significant for most parameters (there was a significant improvement on a delayed memory recall task (*p* = 0.02). This exploratory trial was not powered for clinical outcomes with regard to safety or efficacy.

The REFLECT I trial^39^ was a multicenter, singled blinded RCT that enrolled 54 roll-in patients followed by 204 randomized patients in 2:1 to cerebral protection with TG (n = 141) or control (n = 63). The primary safety outcome was defined per Valve Academic Research Consortium -2 as a composite of all-cause death, stroke, life-threatening or disabling bleeding, acute kidney injury, coronary artery obstruction requiring intervention, major vascular complications, and valve-related dysfunction requiring a repeat procedure. The primary efficacy endpoint was a hierarchical composite of (i) all-cause mortality or any stroke at 30 days; (ii) National Institutes of Health Stroke Scale worsening from baseline to 2 to 5 days postprocedure or Montreal Cognitive Assessment worsening (decrease of 3 points or more from baseline) at 30 days; and (iii) total volume of cerebral ischemic lesions detected by DW-MRI performed 2 to 5 days postprocedure. The primary safety outcome occurred in 21.8% of the TG group, which met the prespecified performance goal of 34.4% (*p* < 0.0001). The primary hierarchical efficacy endpoint was not significantly different between the 2 groups, with a mean score of −5.3 ± 99.8 for TG and 11.8 ± 96.4 for controls (higher score is better; *p* = 0.314). The median total new lesion volume 2 to 5 days post-TAVR also did not differ between the 2 groups (229 mm^3^ vs. 235 mm^3^, *p* = 0.885). Importantly, only 57.3% of patients were reported as having complete cerebral coverage throughout their TAVR procedure due to device movement or interaction between the embolic protection device and the TAVR valve delivery systems. This led to the development of a next-generation TG device (TG3) and the cessation of this trial after enrolling 258 patients. The TG3 device was then evaluated in the REFLECT II trial.

The REFLECT II trial^40^ was a multicenter, single-blinded RCT that was designed to investigate the safety and efficacy of the newer generation TG3 device. This updated device had an enhanced design with a smaller delivery profile (8 Fr instead of 9 Fr) and a reduced filter mesh pore size for the deflection of smaller particles (115 × 145 μm vs. 250 × 250 μm). The study enrolled 220 patients including 41 roll-in and 179 randomized patients (121 TG3 and 58 control subjects) at 18 United States sites. The primary safety and efficacy endpoints were the same as in REFLECT I, and the study again met its safety endpoint (the safety endpoint occurred in 15.9% of patients as compared to the performance goal of 34.4%, *p* < 0.0001). The primary hierarchical efficacy endpoint was again not met and there were no significant differences between the 2 groups in any of the DW-MRI parameters, although this must be interpreted within the context of early termination of the trial leading to a reduced sample size, and imbalanced randomization with a small control group. The study had the same technical difficulties as in REFLECT I (only 59.3% of device patients had complete coverage and 9.6% had device interaction with the TAVR valve platform).

### Embrella

The Embrella Embolic Deflector (EED) (Edwards Lifesciences; Irvine, California) is a deflector device that consists of an oval-shaped nitinol frame covered with a porous polyurethane membrane (100 μm pore size) positioned along the greater curvature of the aorta covering the ostia of the brachiocephalic trunk and left common carotid artery. The device is inserted via the right radial/brachial artery using a 6 Fr delivery system. The EED has been studied in one pilot trial exploring device feasibility and the device subsequently received Conformité Européene-mark approval.^33^ The prospective randomized outcome study in patients undergoing TAVI to examine cerebral ischemia and bleeding complications (PROTAVI-C) trial was a prospective non-randomized study that included 52 patients (41 patients with device and 11 without device as a control group). DW-MRI and transcranial Doppler were performed at baseline, and 7 days and 30 days after TAVR. The use of the EED had no effect on the occurrence and number of new ischemic lesions but was associated with lower lesion volume as compared to the control group. In addition, high-intensity signal rates as evaluated by transcranial Doppler were actually higher in the device group compared to the control, 632 vs. 279 respectively (*p* < 0.001). The study was limited by the low number of patients and lack of randomization. This device is not currently available commercially.

### Embol-X

The EMBOL-X (Edwards Lifesciences, Irvine, California) is a single filter inserted through a mid-sternotomy into the distal part of the ascending aorta with full brain coverage. The device has been studied in a single RCT trial to evaluate the efficacy and safety of the device for patients undergoing transaortic TAVR.^41^ The trial enrolled 30 patients, randomized 1:1 between embolic protection and control. The mean new brain lesion volume was evaluated by DW-MRI at 7 days. The device group showed a non-significant decrease in the presence of new cerebral lesions (57% vs. 69%; *p* = 0.70), and significantly smaller lesion volumes in the supply region of the middle cerebral artery (33 ± 29 vs. 76 ± 67 mm^3^, *p* = 0.04). During the follow-up period, no neurologic event was observed across both groups. This device is limited by its niche application via a sternotomy for patients undergoing transaortic TAVR.

## Synthesizing the Current Evidence for CEPD

The prevention of stroke is an important therapeutic target for patients undergoing TAVR, with the occurrence of stroke occurring within the first 30 days after TAVR being associated with a 6-fold increase in the 30-day mortality.^42^ This has underpinned the development and evaluation of CEPD for adjunctive use during TAVR. Data from the TVT Registry shows CEPD utilization rates were around 6.9% in 2018 and increased to 9.4% in 2019.^43^

**Figure 3:**
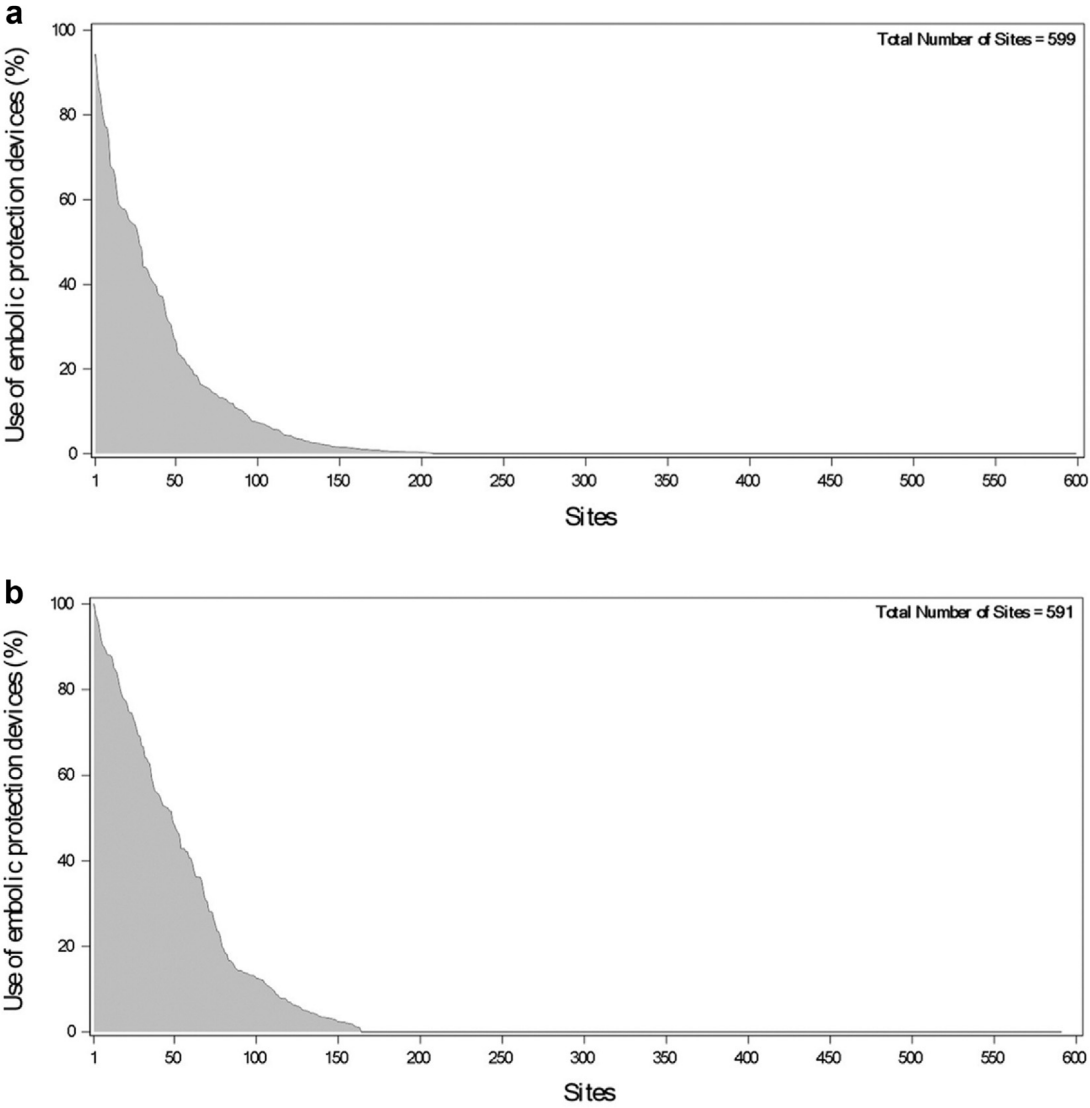
**Wide variation in use of cerebral embolic protection devices across US-hospitals performing transcatheter aortic valve replacement**.

Prior to the PROTECTED-TAVR trial, a meta-analysis of the cerebral protection device trials demonstrated no significant difference in the incidence of stroke across the 6 included RCT trials with a total of 856 randomized patients (relative risk, 0.88; 95% CI, 0.57-1.36; *p* = 0.566; see Figure 3.)^44^ There were also no significant differences in any neuroimaging parameters including the number of new lesions, total lesion volume, or the number of patients seen to have new ischemic lesions. Such analyses were, however, limited by the small number of total randomized patients, the use of different devices within the trials, and the devices potentially being evaluated early in their ‘learning curve’ with operators. The recent PROTECED TAVR trial extended the evidence base for CEPD during TAVR, including significantly more patients than were randomized in all prior trials combined (3000 patients randomized in PROTECTED-TAVR). There was no significant difference in the primary endpoint of stroke within 72 ​hours between the 2 groups, although there was a significant reduction in the rate of disabling stroke with the use of CEPD. Disabling stroke was a non-powered secondary endpoint, and the total number of events was small (8 events in the CEPD arm and 20 in the control arm); this should therefore be considered primarily hypothesis-generating. Such a reduction in disabling stroke would be clinically meaningful to patients and clinicians and is mechanistically plausible if CEPD devices prevent the transit of larger-sized particles to the brain. Given the devastating outcome of disabling stroke, the findings of PROTECTED TAVR warrant further evaluation in ongoing larger-scale trials as planned, and a reduction in disabling stroke might also be associated with reductions in mortality when assessed at the longer-term time points, but this would need to be formally assessed in a randomized trial. CEPD was also seen to be safe in this trial, with no increase in renal injury or vascular complications.^37^

(a) During the entire study period (January 2018 to December 2019). (b) Fourth quarter of 2019. Reproduced with permission from Butala N.M., Makkar R., Secemsky E.A., et al. Cerebral Embolic Protection and Outcomes of Transcatheter Aortic Valve Replacement: Results From the Transcatheter Valve Therapy Registry. Circulation. 2021 Jun 8; 143(23):2229-2240.^45^

**Figure 4:**
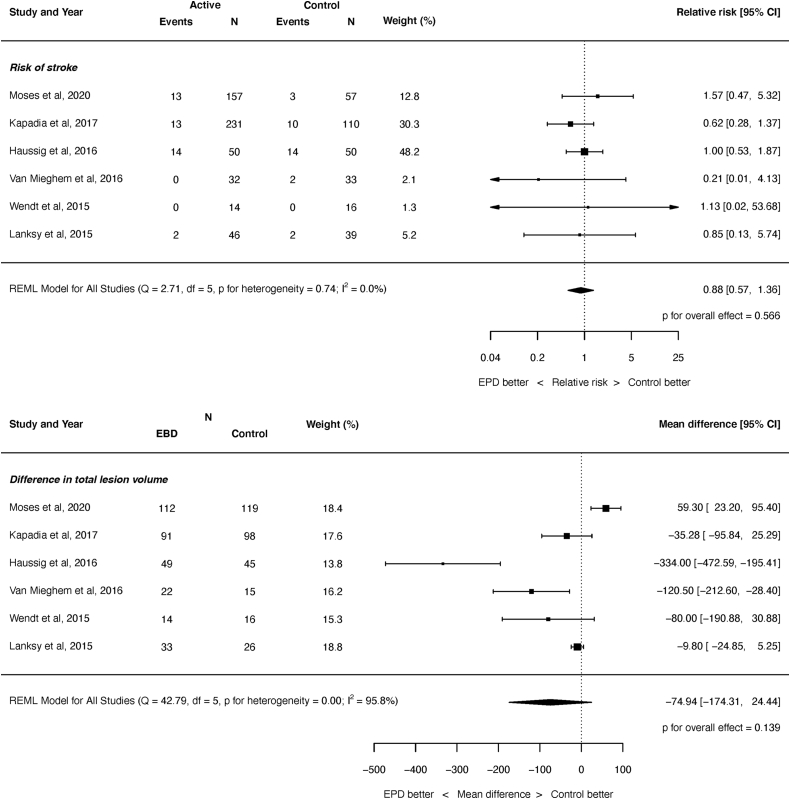
**Meta-analysis of published randomized trials of cerebral embolic protection for the outcomes of stroke (top panel) and total lesion volume (bottom panel)**. Abbreviations: EPD, embolic protection device; REML, residual maximum likelihood.

Reproduced with permission from Ahmad Y., Howard J.P. Meta-Analysis of Usefulness of Cerebral Embolic Protection During Transcatheter Aortic Valve Implantation. Am J Cardiol. 2021 May 1; 146:69-73.^44^

A recent analysis from the Society of Thoracic Surgeons/TVT registry examined CEPD during TAVR across 123,186 patients from 599 sites.^45^ This observational study found wide variation in the use of CEPD across sites, possibly reflecting the limited evidence base for these devices (see Figure 4). Nevertheless, the use of CEPD increased over time (cerebral protection was used in 13% of patients in 2018 and 2019, with increasing utilization time going on, and by the end of 2019, 8% of sites were using cerebral embolic protection in over 50% of cases). The primary analytical method was using an instrumental variable analysis, and this demonstrated no significant benefit from using CEPD on the endpoint of in-hospital stroke (relative risk, 0.90; 95% CI, 0.68-1.13). A secondary analysis using a propensity score-based model did, however, suggest the use of CEPD was associated with a reduction of the odds of in-hospital stroke (OR, 0.82; 95% CI, 0.69-0.97). This nonrandomized analysis is naturally susceptible to residual confounding, although efforts were made to minimize this using the instrumental variable analytical approach.

In another large registry of 108,315 patients undergoing TAVR using the National Inpatient Sample and Nationwide Readmissions Database, CEPD was used in 4380 patients (4.0%).^46^ The analysis showed that the adjusted mortality was lower in patients undergoing TAVR with CEPD (1.3% vs. 0.5%, *p* < 0.01). Furthermore, neurological complications, hemorrhagic stroke, and ischemic stroke (2.2% vs. 1.4%, *p* < 0.01) were also lower in TAVR with CEPD. In a similar analysis of the same dataset, patients who had a stroke after TAVR with CEPD had significantly lower in-hospital mortality than those with stroke after TAVR without CEPD (6.3% vs. 11.8%; *p* = 0.023), as well as shorter length of stay and an increased likelihood of being discharged home.^47^ This raises the possibility that CEPD can prevent more severe and disabling strokes, thereby attenuating stroke-related morbidity and mortality. This is potentially supported by the disabling stroke data from PROTECTED-TAVR, although such analyses are hypothesis-generating only, and necessarily limited by their nonrandomized nature and reliance on administrative claims databases. They also lack adjudication of clinical events and are limited by self-reporting of stroke data.

It can be summarized, therefore, that the use of CEPD during TAVR is safe and feasible, with high rates of successful deployments, quick implantation time, and low access site complication rates as seen in the PROTECTED -TAVR trial. There is no difference in the occurrence of stroke between CEPD and control when assessed in randomized trials, although there was a significant reduction in disabling stroke with the Sentinel CEPD device in the PROTECTED-TAVR trial.^37^

The landscape of this evidence base will continue to change substantially over the coming years (Table 4). The British Heart Foundation Randomised Clinical Trial of Cerebral Embolic Protection in Transcatheter Aortic Valve Implantation (BHF PROTECT-TAVI) trial (Table 4) is currently recruiting in the United Kingdom, with a target sample size of 7730 patients with the same primary endpoint as PROTECTED-TAVR of all strokes occurring within 72 ​hours of the procedure or before hospital discharge. This trial will provide robust data on clinical outcomes across more than 7000 individuals randomized patients, which could potentially shape the evidence behind the CEPD in reducing stroke. The data will also be synthesized in conjunction with the PROTECTED-TAVR data, with an individual patient-level analysis planned of the over 10,000 randomized patients. This will help define the clinical utility of the Sentinel CEPD during TAVR and may also help to identify higher-risk patient subgroups who are more likely to benefit and who might merit further evaluation in dedicated trials.

**Table 4:** Ongoing randomized controlled trial of cerebral embolic protection devices

	BHF PROTECT-TAVI
Design	RCT
Target number of participants	7730
Study arms	Intervention: TAVR with Sentinel No intervention: TAVR without Sentinel
Location	United Kingdom
Inclusion criteria	- Considered to be candidates for TAVR by the clinical team (via any access route where CEPD may be used) - Participant is suitable for treatment with the cerebral embolic protection device in the opinion of the treating physician.
Exclusion criteria	No specific exclusion criteria
Primary endpoint	The incidence of stroke at 72 ​h post-TAVR, or hospital discharge (if sooner)
Secondary endpoints	- The combined incidence of all-cause mortality or non-fatal stroke at 72 ​h post-TAVR, or hospital discharge (if sooner) - Incidence of all-cause mortality at 72 ​h and 12 mo post-TAVR - Incidence of stroke after 72 ​h - Cognitive/disability outcomes - Vascular access site complications - Cost-effectiveness
Funder	British Heart Foundation, Boston Scientific Corporation
Proposed completion date	July 31, 2026

BHF PROTECT- TAVR, the British Heart Foundation randomised clinical trial of cerebral embolic protection in transcatheter aortic valve implantation trial; CEPD, cerebral embolic protection device; RCT, randomized controlled trial; TAVR, transcatheter aortic valve replacement.

## A Pragmatic Clinical Approach

The clinical utility of CEPD will be determined by the aforementioned large-scale, high-quality RCTs powered for the occurrence of stroke.

It is still challenging for treating clinicians to determine whether to offer CEPD to all patients routinely, to no patients at all, or to use these devices selectively in patients they feel to be at high risk of procedural stroke. We do not currently have randomized data to inform which patients truly are at higher risk, and who might be expected to derive benefit from CEPD (there was no benefit of the Sentinel device in any predefined subgroup in the PROTECTED-TAVR trial).

The first step in appropriate patient selection is ensuring the devices are not used in patients with unsuitable anatomy. CEPD should not be used in patients with significant stenoses, dissection, or aneurysms of the brachiocephalic or carotid arteries. Furthermore, caution should be exercised before attempting to employ the devices in patients with significant tortuosity or calcification in the subclavian and arch vessels. There is also a risk that excessive manipulation in the arch and neck vessels attempting to place the device in challenging anatomy could lead to stroke either via atheroembolism or vascular dissection.

Currently, after confirming the anatomic feasibility of placing a CEPD with a detailed analysis of the computerized tomography angiogram, we would advocate weighing the decision on the use of CEPD on the basis of clinical, anatomical, and procedural factors (while awaiting the results of the ongoing RCT). Clinical factors may include patients with prior stroke or TIA, particularly those who may have had a cerebrovascular event as a complication of a prior cardiac catheterization or intervention. Anatomical factors may include patients with a very heavily calcified aortic valve complex, or a bicuspid aortic valve (these are typically heavily calcified and prior analyses have suggested TAVR for bicuspid AS may be associated with an increased risk of stroke compared to TAVR for trileaflet AS),^48^ or patients with severe calcification of their aorta. Finally, procedural factors may include patients in whom increased manipulation across the aortic valve is anticipated, with plans for predilatation and postdilatation of the valve during the TAVR procedure, or a high probability of valve repositioning.^49^ These are pragmatic recommendations, and it should again be emphasized that none of these subgroups were seen to benefit when analyzed in PROTECTED-TAVR.

During the TAVR procedure, an aortic arch angiogram using the already placed pigtail catheter can help with confirming anatomical suitability for CEPD and the optimal sites for basket deployment. We also propose initial wiring with a 300 cm 0.014-inch coronary guidewire after inserting a 6 Fr radial sheath, before opening the device to confirm the absence of prohibitive radial and subclavian anatomy (see Graphical Abstract).

## Conclusions

Stroke after TAVR is a feared and unpredictable outcome and is associated with mortality and significant morbidity; efforts to prevent stroke are therefore an appropriate and important therapeutic target. CEPD are a conceptually sound strategy to protect against stroke during TAVR by preventing embolic debris from reaching the brain. The current evidence base has suggested CEPD is technically feasible and safe to implement, and successfully captures debris in nearly all cases in which it is employed. There is some suggestion that this reduces the number and size of ischemic lesions identified on MRI, but a clear link between prevention of embolic phenomena, reduction in brain lesions on neuroimaging, and improved clinical outcomes have not yet been established. The Sentinel CEPD device did not reduce overall stroke in the 3000 patient PROTECTED-TAVR trial, but there was a significant reduction in disabling stroke. There is hope that future studies, including the ongoing BHF PROTECT-TAVI trial, will more clearly define the clinical utility of current-generation CEPD devices during TAVR, and might help identify higher-risk subgroups that could benefit. Future device development could focus on being able to offer complete cerebral coverage for protection.

## Funding

Mahesh V. Madhavan was supported by a grant from the 10.13039/100000002National Institutes of Health/10.13039/100000050National Heart, Lung, and Blood Institute to 10.13039/100014434Columbia University Irving Medical Center (T32 HL007854).

## Disclosure Statement

John K Forrest is a consultant for Edwards Lifesciences and Medtronic and receives grant support from 10.13039/100006520Edwards Lifesciences, and 10.13039/100004374Medtronic. Raj Makkar has received research grants from 10.13039/100006520Edwards Lifesciences, 10.13039/100000046Abbott, 10.13039/100004374Medtronic, and 10.13039/100008497Boston Scientific; has served as national Principal Investigator for Portico (Abbott) and Acurate (Boston Scientific) U.S. investigation device exemption trials; has received personal proctoring fees from Edwards Lifesciences; and has received travel support from 10.13039/100006520Edwards Lifesciences, 10.13039/100000046Abbott, and 10.13039/100008497Boston Scientific. Martin B. Leon has received research support to his institution from 10.13039/100006520Edwards Lifesciences, 10.13039/100004374Medtronic, 10.13039/100008497Boston Scientific, and 10.13039/100000046Abbott; has served on Advisory Boards for Medtronic, Boston Scientific, Gore, Meril Lifescience, and Abbott; and has served as the Co-Principal Investigator of the PARTNER 3 trial (Edwards Lifesciences, no direct compensation). Alexandra Lansky reports research grants from Sinomed, 10.13039/501100018918Microport, 10.13039/100020297Abiomed, and 10.13039/100008497Boston Scientific, and speaker/consulting fees from Sinomed, Microport, Astra Zeneca, and Medtronic. The other authors had no conflicts to declare.
